# Effects of subsampling on characteristics of RNA-seq data from triple-negative breast cancer patients

**DOI:** 10.1186/s40880-015-0040-8

**Published:** 2015-08-08

**Authors:** Alexey Stupnikov, Galina V Glazko, Frank Emmert-Streib

**Affiliations:** Computational Biology and Machine Learning Laboratory, Faculty of Medicine, Health and Life Sciences, School of Medicine, Dentistry and Biomedical Sciences, Center for Cancer Research and Cell Biology, Queen’s University Belfast, 97 Lisburn Road, Belfast, BT9 7JL UK; Division of Biomedical Informatics, University of Arkansas for Medical Sciences, Little Rock, AR 72205 USA; Computational Medicine and Statistical Learning Laboratory, Department of Signal Processing, Tampere University of Technology, Korkeakoulunkatu 1, Tampere, 33720 Finland

**Keywords:** RNA-seq data, Computational genomics, Statistical robustness, High-dimensional biology, Triple-negative breast cancer

## Abstract

**Background:**

Data from RNA-seq experiments provide a wealth of information about the transcriptome of an organism. However, the analysis of such data is very demanding. In this study, we aimed to establish robust analysis procedures that can be used in clinical practice.

**Methods:**

We studied RNA-seq data from triple-negative breast cancer patients. Specifically, we investigated the subsampling of RNA-seq data.

**Results:**

The main results of our investigations are as follows: (1) the subsampling of RNA-seq data gave biologically realistic simulations of sequencing experiments with smaller sequencing depth but not direct scaling of count matrices; (2) the saturation of results required an average sequencing depth larger than 32 million reads and an individual sequencing depth larger than 46 million reads; and (3) for an abrogated feature selection, higher moments of the distribution of all expressed genes had a higher sensitivity for signal detection than the corresponding mean values.

**Conclusions:**

Our results reveal important characteristics of RNA-seq data that must be understood before one can apply such an approach to translational medicine.

## Background

In recent years, next-generation sequencing technology for generating RNA-seq data has gained considerable interest [1–4] in the biological [5, 6] and biomedical literature [7, 8]. Such data are frequently used, e.g., for identifying alternative splicing, finding differentially expressed genes, or detecting differentially expressed pathways [9–14]. The conventional analysis pipeline for RNA-seq data first maps the reads to genes for a given annotation, resulting in a high-dimensional count vector for each sample. Thereafter, these integer count vectors are normalized and further processed with statistical inference methods. Altering parameters of the preprocessing steps, e.g., aligning procedure, summarization of reads, choice of annotation, and normalization techniques, can change the output of a gene expression analysis drastically. This effect has been studied for different normalization procedures [15].

So far, a major focus has been placed on methods for identifying differentially expressed genes from RNA-seq data [16–18] because such analysis methods that are simpler than, e.g., network-based approaches yet provide meaningful insights into the basic biological functioning of different physiological conditions. Some of these methods assume that the count distribution of individual genes follows a Poisson distribution, whereas others assume a negative binomial distribution for their model. Interestingly, it has been argued that the negative binomial distribution does not perform well under specific conditions [18].

In this study, we carried out an analysis of RNA-seq count distributions for two biological conditions: triple-negative breast cancer (TNBC) samples and TNBC-free samples. The TNBC-free samples corresponded to the same cell types as TNBC samples but were from normal tissue; they formed a control group. For each biological sample, we repeatedly performed a subsampling of mapped reads and thus simulated new samples with a different sequencing depth. For these surrogate gene expression data sets, we studied and compared a variety of properties of their RNA-seq count distributions. We describe the biological data we used for our analysis and the preprocessing steps we applied, and we introduce a procedure, Depth of Sequencing Iterative Reduction Estimator (DESIRE), for subsampling RNA-seq data.

## Methods

### Dataset

The whole data set consists of 6 groups, including a total of 168 samples [19]. We randomly selected four samples of TNBC tumors from the primary tumor group and four samples of healthy breast tissues from TNBC-free group. This selection allowed us to estimate the main statistical entities under investigation. Other samples were not considered in our analysis.

### Data preprocessing

To use RNA-seq data for a gene expression analysis, certain preprocessing steps must be performed. These include alignment of reads, count matrix computation, and normalization.

**Fig. 1:**
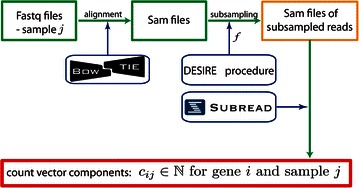
Preprocessing steps of our analysis. Short reads as provided in Fastq files are aligned with Bowtie 2, resulting in Sam files. Application of our method Depth of Sequencing Iterative Reduction Estimator (DESIRE) extracts a defined subsample of size *f*, resulting in updated Sam files. Finally, feature Counts, a function from the *R* subread package, is applied to obtain the count vector for one sample.

After the data were extracted from The Sequence Read Archive [20], we performed the alignment with Bowtie 2 [21] allowing 1 mismatch; human genome version hg38 [22] was taken as the most recent version of reference at the time when the analysis was conducted. To obtain a count vector for a sample (i.e., the number of reads mapped to a gene for all genes), we used the featureCounts function available from the Rsubread package for the R language [23]. During this procedure, the total number of fragments mapped to particular gene positions was summarized. We followed the steps usually implemented for differential gene expression analysis, so various gene isoforms were not of interest. We focused on the gene level for the summarization, not the exon level. The overall process is shown in Fig. 1.

In recent years, a number of different normalization methods have been suggested for the modification of the integer counts for the genes [15]. We preferred “counts per million” (CPM), defined by

1 $$c_{i} = \frac{{N_{i} \times 10^{6} }}{{N_{\text{lib}} }}$$

over “reads per kilobase per million” (RPKM) [24], given by

2 $$c_{i} = \frac{{N_{i} \times 10^{6} }}{{N_{\text{lib}} \times L_{i} }}.$$

Here, *i* corresponds to the index of a gene; *N*_*i*_ is the number of integer counts (reads) for gene *i*; *N*_lib_ is the total number of reads in the library, i.e., the total number of reads per sample; and *L*_*i*_ is the length of an exon (in kilobases).

3 $$N_{\text{lib}} = \sum\limits_{i} {N_{i} }$$

When choosing CPM, we followed the reported argument [18] as the relative difference in expression levels between conditions was the matter of interest.

### Depth of sequencing iterative reduction estimator (DESIRE)

It is commonly accepted that the depth of the sequencing can affect the results of an analysis [25–28]. However, these papers considered only results of a bioinformatics analysis and did not study the details of the count distributions. Another example is the study that addressed the question of the optimal sequencing depth [29].

To study the influence of the sequencing depth on a gene expression analysis, we developed a resampling procedure based on the subsampling of the data. By subsampling, we used only a fraction, *f*, of the total amount of available data in a systematic manner [30]. Another name for such a procedure used in the literature is *m* out of *n* bootstrap, whereas *m* < *n* and the bootstrap samples are drawn without replacement [31]. Our procedure, DESIRE, has the following underlying ideas.

For each biological sample, we drew a number of replicates of a smaller sequencing depth. To accomplish this, a particular portion, *f*, of reads, ranging from 10 to 90%, was randomly drawn from a biological sample without replacement. This process was repeated *R* times resulting in *R* simulated replicates for one simulated sequencing depth *f*. For our analysis, we used *R* = 24 resulting in a total of 240 subsampled data sets for a single biological sample for the 10 different sequencing depths, *f* = {0.1,…, 0.9, 1.0}.

The specific value of *R* is not crucial. However, if it is large, the computational complexity would increase without resulting in significant improvements in the statistical estimates of our analysis. On the other hand, values of *R* much lower than 24 potentially result in unstable results. The particular number of *R* = 24 considered the number of nodes in our computer cluster available for our analysis.

**Fig. 2:**
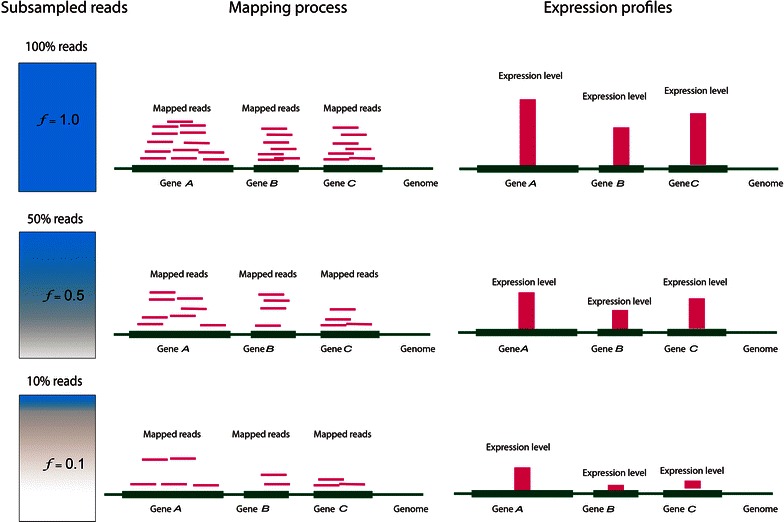
Overview of the DESIRE procedure.

**Fig. 3:**
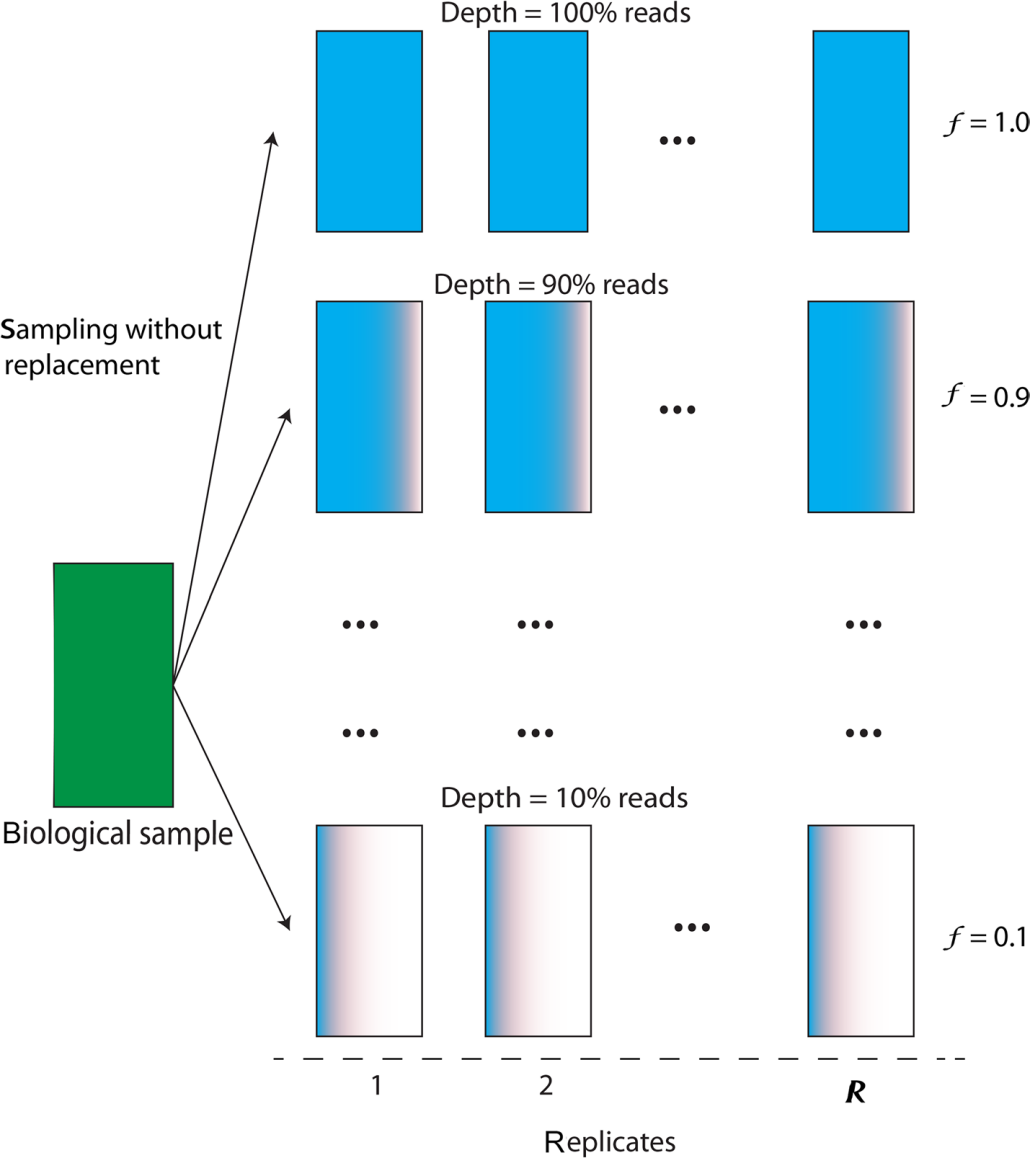
Generation of *R* replicates for a given sequencing depth using only a fraction, *f*, of the original data in a biological sample. Hence, each of the *R* generated data sets is a subsample of the original biological sample.

A schematic overview of the DESIRE procedure is shown in Figs. 2 and 3. It is important to note that the simulated sequencing death, *f*, refers to all reads of the genome and not to the reads of a single gene. In this way, DESIRE simulates actual biological experiments conducted for a smaller sequencing depth. If we draw *f* reads for each gene independently, the resulting samples would not correspond to results produced by next-generation sequencing technology, e.g., on an Illumina platform.

We calculated the count vectors using Entrez annotation from Bioconductor, database org.Hs.e.g.db2.9.0, which consisted of 23,648 (protein-coding and -noncoding) genes [32].

## Results

The purpose of our study was to learn about the influence of the sequencing depth on inferred biological results. For this reason, we investigated 4 layers of complexity. First, we compared differences between an explicit subsampling of reads and a direct scaling of count matrices. The results from this analysis demonstrated that a subsampling via DESIRE was necessary to obtain realistic surrogates of sequencing experiments with a smaller sequencing depth. Second, we studied the absolute expression of genes and their growth. Third, we investigated the growth rate of the number of expressed genes. Fourth, we analyzed differences in the distributional shape of expressed genes between TNBC patients and TNBC-free patients. For each of these analysis steps, we used data generated by the DESIRE procedure.

### Differences between subsampling of reads and direct scaling of count matrices

Our first analysis investigated differences between a subsampling of reads via the DESIRE procedure and a direct scaling of count matrices. The results of this analysis justified our approach for the following sections.

The basic idea of DESIRE is to draw randomly aligned reads, as provided by a Sam file, and create a new auxiliary Sam file corresponding to a new sequencing experiment with a smaller sequencing depth. We compared this with a direct scaling of count matrices, whereas the scaling was obtained by multiplying the components of the count matrices, *c*_*ij*_, with a constant factor *f* that corresponds to the simulated sequencing depth because

4 $$\frac{{{\text{Total}}\;{\text{number}}\;{\text{of}}\;{\text{scaled}}\;{\text{counts}}}}{{{\text{Total}}\;{\text{number}}\;{\text{of}}\;{\text{counts}}}} = \frac{{\sum\nolimits_{i,j} {f \times c_{ij} } }}{{\sum\nolimits_{i,j} {c_{ij} } }} = f$$

Hence, this simple scaling of a count matrix resulted in the desired simulated sequencing depth for a sample.

**Fig. 4:**
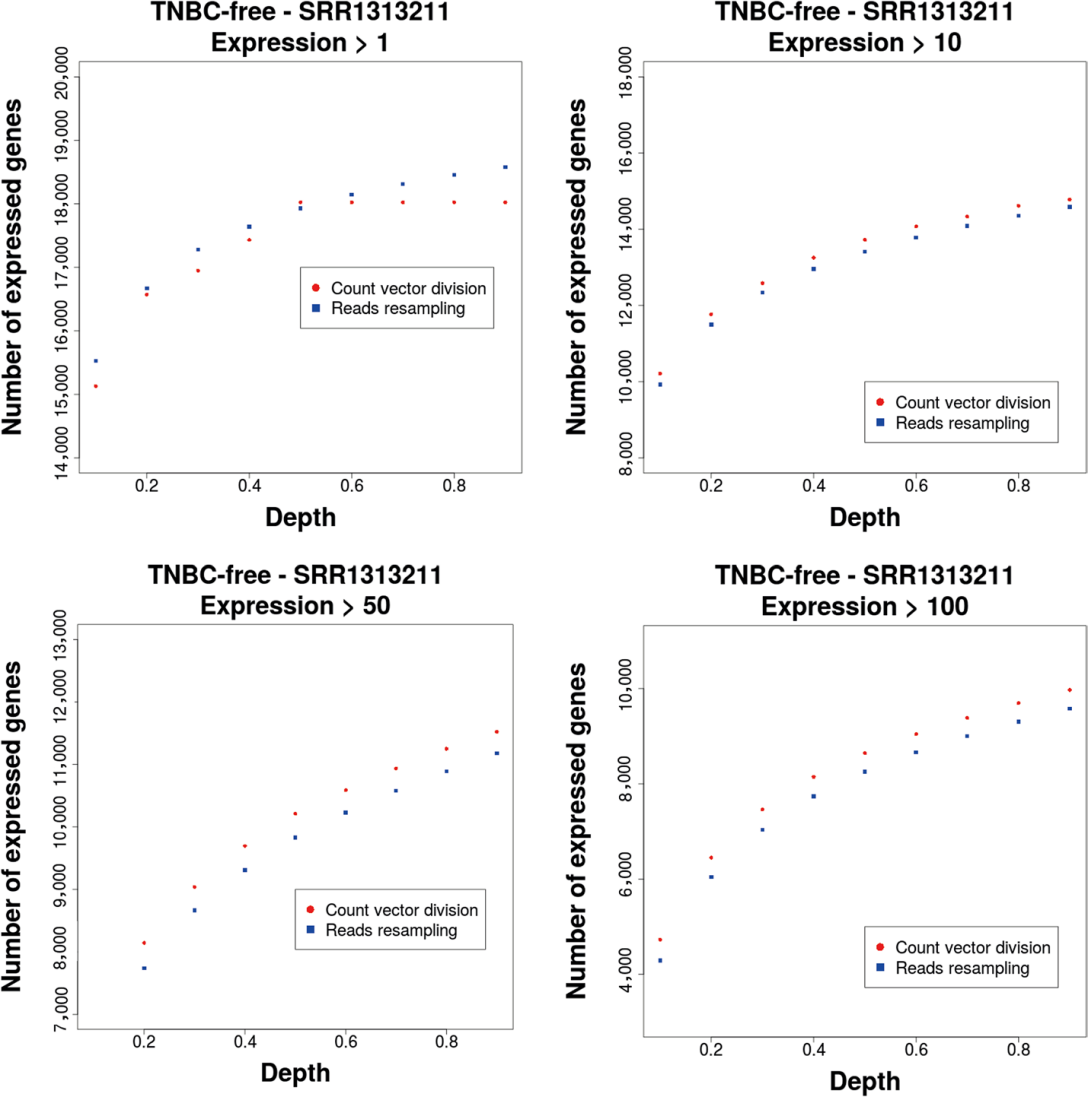
Comparison of the subsampling of reads via the DESIRE procedure (*blue*) and a direct scaling of count matrices (*red*). The obtained numbers of expressed genes depending on the sequencing depth for four different threshold parameters (1, 10, 50, 100) are shown.

For one TNBC-free sample (SRR1313211), the difference between counts obtained via our DESIRE procedure and the direct scaling method of count matrices is shown in Fig. 4. Specifically, the number of expressed genes (*Y* axis), depending on the sequencing depth *f* (*X* axis), for different values of a threshold parameter is presented in Fig. 4. By the number of expressed genes, we meant the number of genes that have a short read count *c*_*ij*_ of ⊝ϵ{1, 10, 50, 100} or larger, i.e., *c*_*ij*_ ≥ ⊝, where ⊝ is the threshold parameter. All results are for raw count values, not normalized values, and each dot corresponds to the result from one data set.

For all threshold values and all sequencing depths that we investigated, there were distinct differences between the two approaches (Fig. 4). Similar results were also observed in other patient samples (not shown). From these results, we concluded that the computationally efficient shortcut via a direct scaling of count matrices did not lead to the same results as the DESIRE procedure. Hence, the scaled count matrices did not correspond to sequencing experiments with a smaller sequencing depth but had an unclear biological interpretation. For this reason, the DESIRE procedure needs to be used for simulating realistic sequencing experiments because only in this way do the resulting data have a clear interpretation in biological terms. In the following sections, we used the DESIRE procedure for this purpose.

We would like to note that neither our statistic, the number of expressed genes, nor the specific threshold ⊝ was crucial for our conclusion, but other statistics led to similar results. For our following analysis, it was important only that there was a difference but not how each individual measure was affected. However, we thought that for particular measures that were used, e.g., as test statistic for hypothesis tests or distance metrics for clustering, it might be interesting to quantify these differences more specifically.

### Absolute expression of genes

**Fig. 5:**
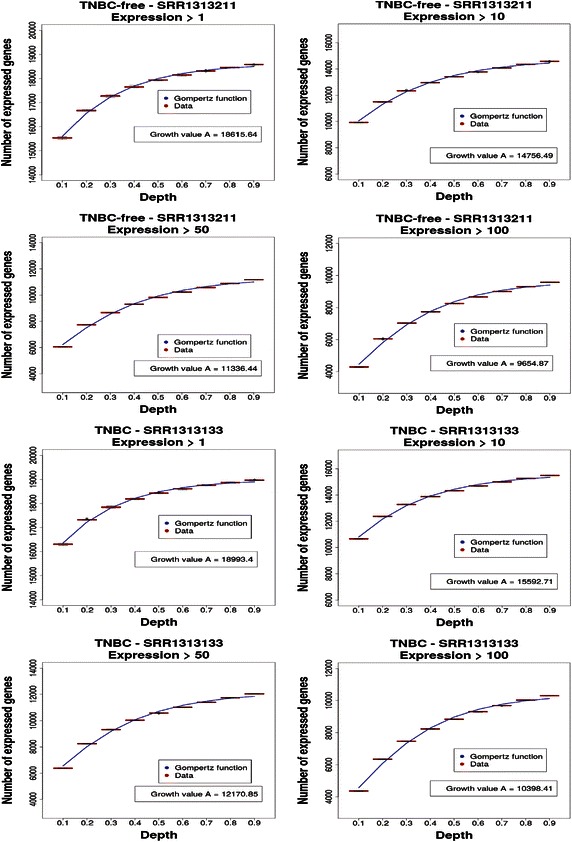
Triple-negative breast cancer (TNBC)-free sample SRR1313211 and TNBC sample SRR1313133. The number of expressed genes for different filtering thresholds (1, 10, 50, 100) depends on the sequencing depth. The *blue curves* correspond to fitted Gompertz functions. All results are for raw (unnormalized) count values.

In this analysis, we studied the influence of the sequencing depth on the number of expressed genes. The results for a TNBC-free patient (SRR1313211) and a TNBC patient (SRR1313133), exemplary for all samples studied, are shown in Fig. 5; the number of expressed genes (*Y* axis), depending on the sequencing depth *f* (*X* axis) for different values of a threshold parameter ⊝ϵ{1, 10, 50, 100}, are also presented. All results are for raw count values, not normalized values, and for each sequencing depth *f*, we generated *R* = 24 subsampled data sets for which box plots are shown.

The first impression of the overall behavior was intuitive because the larger was the sequencing depth, the higher was the probability to obtain at least ⊝ reads for a gene, if it was expressed. Less intuitive was the fact that for all samples and all thresholds, there was no saturation in the number of expressed genes, but this number continued to grow, which suggests that even the maximally available sequencing depth was not sufficient to achieve a saturation of the measurements. In addition, this pointed to possible errors in either the sequencing or the alignment of reads because it was biologically implausible to assume that almost all 23,648 genes considered by our analysis were actually expressed for ⊝ = 1 (Fig. 5). This may open the possibility to quantify such errors statistically.

From the obtained results in Fig. 5 and the results from 6 further samples that looked qualitatively similar (not shown), we attempted to estimate the optimal sequencing depth in the following two ways using the available sequencing depth of the samples used for our analysis (TNBC samples: 34974017, 46677107, 17574408, and 24440340; TNBC-free samples: 25900791, 43454785, 31426867, and 33517581). Estimator (I)—average sequencing depth: the first estimator centers on average properties of our samples. Given that the average number of short reads per sample was 32,245,737 ± 9,710,593 (averaged over the 8 samples) and the fact that none of the growth curves saturated, we estimated that the average number of reads necessary for a saturation must be larger than 32,245,737. Estimator (II)—individual sequencing depth: the second estimator centers on the individual samples. The largest sequencing depth of our samples was 46,677,107, and even this sample did not lead to a saturating growth. Hence, a conservative estimate requires an individual sequencing depth larger than 46,677,107.

The variability of all results, e.g., the interquartile range (IQR) of the box plots, was in general quite small. However, for larger ⊝ values, the IQR was even further decreased, which showed that the estimation for the number of expressed genes was even more stable for larger expression threshold values, corresponding to a more stringent filtering for expressed genes.

For a quantitative comparison between the TNBC and TNBC-free patient samples, we compared the mean of median values of the number of expressed genes, for different sequencing depths *f*, to test the null hypothesis:

5 $$H_{0|f} :{\text{mean(median}}_{\text{TNBC|f}} )\;{ = }\;{\text{mean(median}}_{\text{TNBC-free|f}} )$$

by a two-sample *t* test. Each comparison was based on 4 samples per condition. Here, for instance, median_TNBC|f_ indicates the conditional median value of TNBC patients, conditioned on the sequencing depth *f*. The other conditional symbols have a similar meaning.

The results of these hypothesis tests are shown in Table 1. For a significance level of α = 0.05, only one result for a left-sided test was significant for *f* = 0.1. However, all other *P* values from the left-sided comparison were approximately 5%, indicating a tendency of being different but not significantly. This is plausible because we know that the samples from TNBC and TNBC-free patients corresponded to two different physiological conditions but that these differences affected some, but not all, biological processes, e.g., the hallmarks of cancer [33]. Hence, if samples are compared as a whole, as in our case, using only the mean of the medians of the number of expressed genes as a test statistic and not adjusting for different types of biological processes, e.g., using information from the gene ontology database [34], this signal is too weak to be detected. On the other hand, we found that the number of expressed genes in TNBC patients was smaller than that in TNBC-free patients because there was a clear asymmetry between the left- and right-sided *P* values, always leading to the relation

**Table 1:** Results of two-sample *t* tests comparing the total number of expressed genes for various sequencing depths

Depth	*P* value, two-sided	*P* value, left-sided	*P* value, right-sided
0.1	0.096,85	0.048,45	0.951,57
0.2	0.125,02	0.062,51	0.937,49
0.3	0.124,18	0.062,10	0.937,91
0.4	0.123,61	0.061,81	0.938,19
0.5	0.118,90	0.059,45	0.940,55
0.6	0.128,56	0.064,28	0.935,72
0.7	0.145,83	0.072,92	0.927,08
0.8	0.161,76	0.080,88	0.919,12
0.9	0.155,24	0.077,62	0.922,38

The total number of expressed genes for various sequencing depths is shown in Fig. 5.

6 $${\text{p}}\;{\text{value}}_{\text{left-sided}} \ll {\text{p}}\;{\text{value}}_{\text{right-sided}}$$

This relation indicated that, on average, there were fewer genes expressed in TNBC patients than in the corresponding control samples, independent of the sequencing depth.

### Growth rate of the number of expressed genes

Next, we compared the growth of the number of expressed genes depending on the sequencing depth (Fig. 5). For this reason, we fitted Gompertz growth functions [35] given by

7 $$f(x) = a\exp \left( { - b\exp \left( { - cx} \right)} \right)$$

Here *a*, *b*, and *c* are parameters of the Gompertz function to be fitted and *c* is called the *growth rate*. For our quantitative comparison, we used the fitted values of *c*.

We used Gompertz growth functions because the number of (expressed) genes of an organism was limited and, hence, so was the increase in the number of genes having more than a certain threshold needed to saturate. Growth curves, such as the Gompertz function or the logistic function [36, 37], have the natural constraint of being limited from above and, hence, provide a natural choice for a constrained regression function. Table 2 shows the growth rates and their standard deviations for all 8 samples and the 4 threshold values, ⊝ϵ {1, 10, 50, 100}.

**Table 2:** Fitted growth factor values and standard deviations for the Gompertz functions

Depth	Sample	Condition	Growth rate (SD)
1	SRR1313137	TNBC	18,645.168 (71.428)
1	SRR1313135	TNBC	19,218.431 (51.492)
1	SRR1313134	TNBC	18,885.949 (71.780)
1	SRR1313133	TNBC	18,993.399 (58.036)
1	SRR1313211	TNBC-free	18,615.636 (77.401)
1	SRR1313214	TNBC-free	18,726.438 (82.876)
1	SRR1313219	TNBC-free	18,286.856 (82.281)
1	SRR1313220	TNBC-free	18,930.056 (85.636)
10	SRR1313137	TNBC	14,904.344 (144.082)
10	SRR1313135	TNBC	16,096.457 (121.821)
10	SRR1313134	TNBC	15,053.704 (139.567)
10	SRR1313133	TNBC	15,592.711 (155.879)
10	SRR1313211	TNBC-free	14,756.491 (152.976)
10	SRR1313214	TNBC-free	14,740.701 (158.437)
10	SRR1313219	TNBC-free	13,971.554 (166.406)
10	SRR1313220	TNBC-free	15,280.019 (143.239)
50	SRR1313137	TNBC	11,532.85 (163.987)
50	SRR1313135	TNBC	12,782.861 (162.683)
50	SRR1313134	TNBC	11,211.735 (166.459)
50	SRR1313133	TNBC	12,170.85 (205.805)
50	SRR1313211	TNBC-free	11,336.443 (195.497)
50	SRR1313214	TNBC-free	11,514.378 (174.158)
50	SRR1313219	TNBC-free	10,577.339 (166.987)
50	SRR1313220	TNBC-free	11,654.075 (193.834)
100	SRR1313137	TNBC	9,983.466 (176.904)
100	SRR1313135	TNBC	11,235.174 (168.548)
100	SRR1313134	TNBC	9,634.957 (198.902)
100	SRR1313133	TNBC	10,398.413 (194.712)
100	SRR1313211	TNBC-free	9,654.874 (175.648)
100	SRR1313214	TNBC-free	9,898.436 (162.635)
100	SRR1313219	TNBC-free	9,089.057 (145.683)
100	SRR1313220	TNBC-free	9,947.798 (162.972)

*TNBC* triple-negative breast cancer and *SD* standard deviation.

From a visual inspection, there were only slight differences between the different conditions. For this reason, we quantified the results to test the null hypothesis that there was no difference in the values of the growth rates, i.e.,

8 $$H_{0|f} :{\text{mean(}}c_{\text{TNBC|f}} )\;{ = }\;{\text{mean(}}c_{\text{TNBC-free|f}} ),$$

for depth by a two-sample *t* test. Again, each comparison was based on 4 samples per condition.

To identify direction-specific effects, we also performed hypothesis tests for two-sided, left-sided, and right-sided comparisons. The results of these hypothesis tests are shown in Table 3. Overall, for a significance level of *α* = 0.05, none of these hypothesis tests was significant. However, the right-sided *P* values were not much larger than 0.05, hinting at a tendency in the data to be different, like the comparison of the median number of expressed genes above.

**Table 3:** Results from comparing the growth rates of the fitted Gompertz functions for TNBC and TNBC-free patients

Depth	*P* value, two-sided	*P* value, left-sided	*P* value, right-sided
1	0.151,2	0.924,4	0.075,6
10	0.107,2	0.946,4	0.053,6
50	0.179,5	0.910,2	0.089,8
100	0.157,4	0.921,3	0.078,7

The Gompertz functions for TNBC and TNBC-free patients are shown in Table 2. Abbreviation as in Table 2.

A normalization of the data does not remove the growth property observed in Fig. 5, but normalized data exhibit qualitatively the same behavior. For ⊝ = 1, this was obvious because the normalization led to a scaling of the data without changing the zero values. For ⊝ > 1, it was less intuitive but followed from our numerical analysis (results not shown).

### Distributional shape of expressed genes

Last, we studied the distributional shape of expressed gene values (and not of their numbers) by estimating individually for each parameter configuration its mean value, variance, skewness, and kurtosis. Here, we mean the distribution over all genes within a sample, and not the count distribution of individual genes across samples. Because every distribution with existing moments was fully characterized by all of its moments, either via its moment-generating function or via its probability generating function [38, 39], our analysis was an approximation of the distributional shape because we limited our focus to 4 dimensions.

**Fig. 6:**
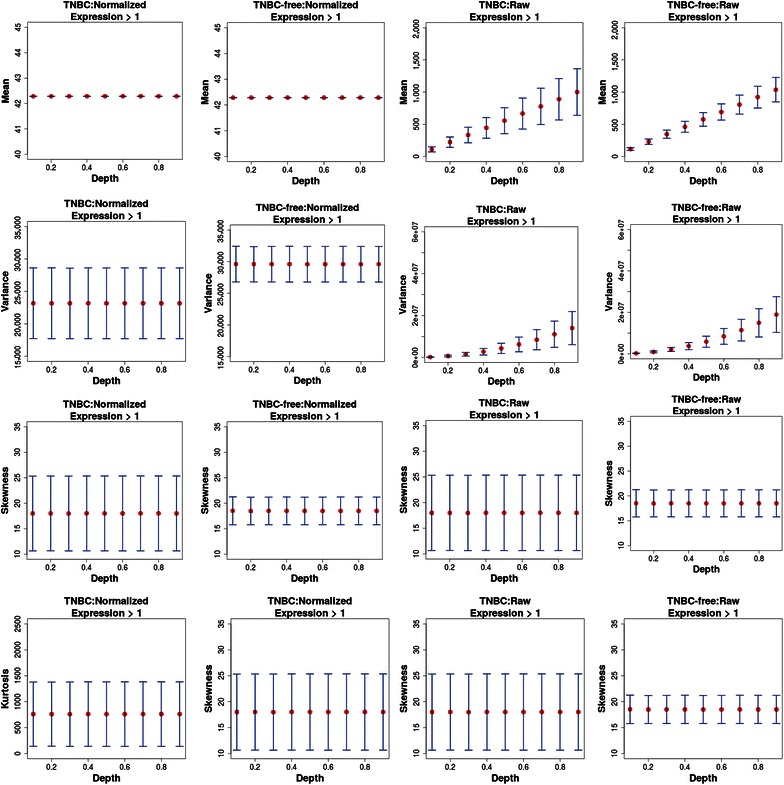
Results for the 4 moments: mean, variance, skewness, and kurtosis (*rows*). *Columns 1* and *2*: normalized data; *Columns 3* and *4*: raw data; *Columns 1* and *3*: TNBC patients; *Columns 2* and *4*: TNBC-free patients.

Specifically, for each condition (TNBC versus TNBC-free) and each sequencing depth (*f* ϵ {1, 10, 50, 100}), we generated *R* = 24 data sets, giving a total of 432 data sets, and applied the expression threshold ⊝ = 1 to each data set as a filter. In the following analysis, we distinguished between CPM normalized and raw (unnormalized) data by estimating the mean, variance, skewness, and kurtosis of the distributions of expression values of the genes. The results of this analysis are shown in Fig. 6 and Tables 4 and 5, which include results for raw (unnormalized) data in Columns 3 and 4. The first observation from Fig. 6 is that a normalization of the data was absolutely necessary to obtain stable results across different sequencing depths. This is clearly visible for the mean and variance values because they showed increasing values for larger sequencing depths. In this respect, even a simple CPM normalization counterbalanced this effect, leading to stable expression patterns across different sequencing depths. This also illustrated that the choice of normalization method affected the statistical properties of a distribution and the results of statistical inference significantly, such as differential gene expression analysis, which was also observed [15]. From a visual comparison of the moments for TNBC and TNBC-free patients, we observed clear differences between the variance, less clear differences for the kurtosis and neutral differences for the mean and skewness. For a quantification of the comparison between the moments for TNBC and TNBC-free patients, we tested the following null hypothesis by a two-sample *t* test:

9 $$H_{0|f} :{\text{mean(}}m_{\text{TNBC|f}} )\;{ = }\;{\text{mean(}}m_{\text{TNBC-free|f}} ),$$

for depth *f* and *m*ϵ{mean, variance, skewness, kurtosis}, indicating the four moments we studied. Each comparison was based on nine samples per condition because we pooled the median values across the different sequencing depths for each condition and each measure *m*. The results of this analysis are shown in Table 6. Overall, the mean values were essentially undistinguishable (with *P* values of approximately 1.0) but the other three moments were significantly different at a two-sided significance level of α = 0.05. Specifically, for the kurtosis and skewness, the left-sided tests were significant; for the variance, the right-sided test was significant. That means that for kurtosis and skewness, the values of the moments were higher in TNBC-free patients than in TNBC patients, whereas for variance, these values were lower. This result is interesting because, commonly, a disease is associated with instability or disorder, but a decreasing variance suggested less variability in the expression values of the genes.

**Table 4:** Moments for TNBC-free patients

Depth	Mean (SD)	Variance (SD)	Skewness (SD)	Kurtosis (SD)
0.1	42.29 (0)	29,616.28 (2,817.52)	18.51 (2.75)	599.44 (222.29)
0.2	42.29 (0)	29,588.5 (2,790.57)	18.48 (2.71)	597.09 (219.38)
0.3	42.29 (0)	29,600.99 (2,803.23)	18.50 (2.71)	598.63 (219.27)
0.4	42.29 (0)	29,602.40 (2,819.85)	18.51 (2.72)	598.92 (220.32)
0.5	42.29 (0)	29,592.22 (2,807.72)	18.49 (2.71)	597.70 (218.81)
0.6	42.29 (0)	29,597.76 (2,805.76)	18.50 (2.71)	598.30 (219.30)
0.7	42.29 (0)	29,599.37 (2,807.41)	18.51 (2.72)	599.09 (220.33)
0.8	42.29 (0)	29,595.86 (2,805.56)	18.51 (2.72)	598.86 (220.13)
0.9	42.29 (0)	29,593.61 (2,806.58)	18.50 (2.71)	598.45 (219.59)

The moments for TNBC-free patients are also presented in Fig. 6. Abbreviations as in Table 2.

**Table 5:** Moments for TNBC patients

Depth	Mean (SD)	Variance (SD)	Skewness (SD)	Kurtosis (SD)
0.1	42.29 (0)	23,176.53 (5446.28)	17.99 (7.33)	759.6 (618.96)
0.2	42.29 (0)	23,172.85 (5454.44)	18.01 (7.33)	760.33 (618.23)
0.3	42.29 (0)	23,155.44 (5437.48)	17.99 (7.34)	758.8 (620.44)
0.4	42.29 (0)	23,169.84 (5448.77)	18.02 (7.35)	761.69 (621.23)
0.5	42.29 (0)	23,165.79 (5448.29)	18.01 (7.34)	760.62 (620.49)
0.6	42.29 (0)	23,166.48 (5449.83)	18.01 (7.35)	760.84 (621.57)
0.7	42.29 (0)	23,168.39 (5451.98)	18.02 (7.35)	761.56 (621.74)
0.8	42.29 (0)	23,165.7 (5448.48)	18.02 (7.35)	761.45 (621.81)
0.9	42.29 (0)	23,164.67 (5448.95)	18.01 (7.35)	761.16 (621.53)

The moments for TNBC patients are also presented in Fig. 6. Abbreviations as in Table 2.

**Table 6:** Results from pooled (across different sequencing depths) two-sample *t* tests for the 4 moments of the gene expression distributions

Moment	*P* value, two-sided	*P* value, left-sided	*P* value, right-sided
Mean	1.0	1.0	1.0
Variance	2.238800e−11	1.000000e + 00	1.119400e−11
Kurtosis	1.966589e−04	9.832945e−05	9.999017e−01
Skewness	6.808568e−03	3.404284e−0.3	9.965957e−01

The 4 moments of the gene expression distributions are shown in Fig. 6.

## Discussion

In this paper, we studied various effects of differing sequencing depth on distributional aspects of gene expression data obtained from RNA-seq experiments. From our analysis, we found 3 main results.

1. The subsampling of RNA-seq data gave biologically realistic simulations of next-generation sequencing experiments with smaller sequencing depth, but a direct scaling of count matrices did not. This is an important finding because, first of all, it demonstrated that the conceptually simpler and computationally more efficient approach of a direct scaling of count matrices led to data sets with an unclear biological interpretation. This is of course a major problem because whatever results were obtained from such data sets, e.g., using them for identifying differentially expressed genes, the meaning is at best unclear and possibly even uninterruptable in the sense that replicated next-generation sequencing experiments would not result in data with such a characteristic.
2. To obtain saturating results, we estimated an average sequencing depth of >32 million reads and an individual sequencing depth of >46 million reads. The literature gives context-specific suggestions. For instance, for detecting rare transcripts in human, >200 million paired-end reads should be used, and for the accurate quantification of genes across the entire expression range, >80 million reads per sample should be used [29, 40]. However, for the identification of differentially expressed genes, 36 million reads per sample may be sufficient [29].
  For future studies, it would be interesting to derive improved bounds for optimal sequencing depths with respect to two complementary aspects. The first aspect involves distinguishing different application domains because the optimal sequencing depth is likely to depend on the bioinformatics analysis. For gene expression data from DNA microarray experiments, such differences have already been known for, e.g., methods identifying differentially expressed genes and methods for identifying differentially expressed gene sets [41–43]. Second, in this study, we considered only simple statistical estimators for the optimal sequencing depth; however, more elaborate approaches are possible, e.g., by exploiting the results from the fitted growth curves.
3. For an abrogated feature selection, i.e., using all expressed genes that have read counts of ⊝ = 1 or larger, the higher moments of the distribution of expressed genes showed a much better sensitivity for the signal detection of differing phenotypic conditions than the corresponding mean values (Table 6). This could be further explored by designing statistical tests that use such higher moments as a test statistic. A potential advantage of such tests over, e.g., the conventional mean-based tests such as a *t* test or ANOVA could be a reduced need in sample size, as suggested by our results. However, this requires a further detailed analysis.

## Conclusions

The subsampling of RNA-seq data allows us to explore important aspects of gene expression data. These must be understood before such high-throughput data types can be used for applications in translational medicine.

## References

[1] McGettigan PA (2013). Transcriptomics in the RNA-seq era. Curr Opin Chem Biol.

[2] Marguerat S, Bähler J (2010). RNA-seq: from technology to biology. Cell Mol Life Sci.

[3] Metzker ML (2009). Sequencing technologies–the next generation. Nat Rev Genet.

[4] Wang Z, Gerstein M, Snyder M (2009). RNA-Seq: a revolutionary tool for transcriptomics. Nat Rev Genet.

[5] Mortazavi A, Williams BA, McCue K, Schaeffer L, Wold B (2008). Mapping and quantifying mammalian transcriptomes by RNA-seq. Nat Methods.

[6] Peng Z, Cheng Y, Tan BC, Kang L, Tian Z, Zhu Y (2012). Comprehensive analysis of RNA-Seq data reveals extensive RNA editing in a human transcriptome. Nat Biotechnol.

[7] Beane J, Vick J, Schembri F, Anderlind C, Gower A, Campbell J (2011). Characterizing the impact of smoking and lung cancer on the airway transcriptome using RNA-Seq. Cancer Prev Res.

[8] Sinicropi D, Qu K, Collin F, Crager M, Liu ML, Pelham RJ (2012). Whole transcriptome RNA-Seq analysis of breast cancer recurrence risk using formalin-fixed paraffin-embedded tumor tissue. PLoS One.

[9] Anders S, Huber W (2010). Differential expression analysis for sequence count data. Genome Biol.

[10] Rahmatallah Y, Emmert-Streib F, Glazko G (2014). Comparative evaluation of gene set analysis approaches for RNA-Seq data. BMC Bioinform.

[11] Nicolae M, Mangul S, Mandoiu II, Zelikovsky A (2011). Estimation of alternative splicing isoform frequencies from RNA-Seq data. Algorithm Mol Biol.

[12] Robinson MD, Oshlack A (2010). A scaling normalization method for differential expression analysis of RNA-seq data. s.

[13] Trapnell C, Pachter L, Salzberg SL (2009). TopHat: discovering splice junctions with RNA-Seq. Bioinformatics.

[14] Wang L, Feng Z, Wang X, Wang X, Zhang X (2010). DEGseq: an R package for identifying differentially expressed genes from RNA-seq data. Bioinformatics.

[15] Dillies MA, Rau A, Aubert J, Hennequet-Antier C, Jeanmougin M, Servant N (2013). A comprehensive evaluation of normalization methods for illumina high-throughput RNA sequencing data analysis. Brief Bioinform.

[16] Wu H, Wang C, Wu Z (2013). A new shrinkage estimator for dispersion improves differential expression detection in RNA-seq data. Biostatistics.

[17] Robinson MD, McCarthy DJ, Smyth GK (2010). edgeR: a Bioconductor package for differential expression analysis of digital gene expression data. Bioinformatics.

[18] Law C, Chen Y, Shi W, Smyth G (2014). Voom: precision weights unlock linear model analysis tools for RNA-seq read counts. Genome Biol.

[19] Varley KE, Gertz J, Roberts BS, Davis NS, Bowling KM, Kirby MK (2014). Recurrent read-through fusion transcripts in breast cancer. Breast Cancer Res Treat.

[20] Leinonen R, Sugawara H, Shumway M (2010). International Nucleotide Sequence Database Collaboration. The sequence read archive. Nucleic Acids Res.

[21] Langmead B, Salzberg SL (2012). Fast gapped-read alignment with Bowtie 2. Nat Methods.

[22] Karolchik D, Barber GP, Casper J, Clawson H, Cline MS, Diekhans M (2014). The UCSC genome browser database: 2014 update. Nucleic Acids Res.

[23] Liao Y, Smyth GK, Shi W (2013). featureCounts: an efficient general purpose program for assigning sequence reads to genomic features. Bioinformatics.

[24] Mortazavi A, Williams BA, McCue K, Schaeffer L, Wold B (2008). Mapping and quantifying mammalian transcriptomes by RNA-Seq. Nat Methods.

[25] Fumagalli M (2013). Assessing the effect of sequencing depth and sample size in population genetics inferences. PLoS One.

[26] Rapaport F, Khanin R, Liang Y, Pirun M, Krek A, Zumbo P (2013). Comprehensive evaluation of differential gene expression analysis methods for RNA-seq data. Genome Biol.

[27] Robinson DG, Storey JD (2014). subSeq: determining appropriate sequencing depth through efficient read subsampling. Bioinformatics.

[28] Liu Y, Zhou J, White KP (2014). RNA-seq differential expression studies: more sequence or more replication?. Bioinformatics.

[29] Sims D, Sudbery I, Ilott NE, Heger A, Ponting CP (2014). Sequencing depth and coverage: key considerations in genomic analyses. Nat Rev Genet.

[30] Politis DN, Romano JP, Wolf M (1999). Subsampling Springer series in statistics.

[31] Bickel PJ, Gotze F, van Zwet W (1997). Resampling fewer than n observations: gains, losses and remedies for losses. Statist Sinica..

[32] Gentleman RC, Carey VJ, Bates DM, Bolstad B, Dettling M, Dudoit S (2004). Bioconductor: open software development for computational biology and bioinformatics. Genome Biol.

[33] Hanahan D, Weinberg RA (2000). The hallmarks of cancer. Cell.

[34] Ashburner M, Ball CA, Blake JA, Botstein D, Butler H, Cherry JM (2000). Gene ontology: tool for the unification of biology. Gene ontology consortium. Nat Genet.

[35] Laird AK (1964). Dynamics of tumour growth. Br J Cancer.

[36] Emmert-Streib F (2013). Structural properties and complexity of a new network class: Collatz step graphs. PLoS One.

[37] Harrell FE (2001). Regression modeling strategies.

[38] Casella G, Berger RL (2002). Statistical inference.

[39] Feller W (1968). An introduction to probability theory and its applications.

[40] Tarazona S, Garcia-Alcalde F, Dopazo J, Ferrer A, Conesa A (2011). Differential expression in RNA-seq: a matter of depth. Genome Res.

[41] Emmert-Streib F, Tripathi S, de Matos Simoes R (2012). Harnessing the complexity of gene expression data from cancer: from single gene to structural pathway methods. Biol Direct.

[42] Hung JH, Yang TH, Hu Z, Weng Z, DeLisi C (2012). Gene set enrichment analysis: performance evaluation and usage guidelines. Brief Bioinform.

[43] Steinhoff C, Vingron M (2006). Normalization and quantification of differential expression in gene expression microarrays. Brief Bioinform.

